# Extracting Tissue Optical Properties and Detecting Bruised Tissue in Pears Quickly and Accurately Based on Spatial Frequency Domain Imaging and Machine Learning

**DOI:** 10.3390/foods12020238

**Published:** 2023-01-04

**Authors:** Shengqiang Xing, Jiaming Zhang, Yifeng Luo, Yang Yang, Xiaping Fu

**Affiliations:** 1 School of Information Science and Engineering, Zhejiang Sci-Tech University, Hangzhou 310018, China; 2 Key Laboratory of Transplanting Equipment and Technology of Zhejiang Province, Hangzhou 310018, China

**Keywords:** spatial frequency domain imaging (SFDI), optical properties, absorption, reduced scattering, long short-term memory (LSTM)

## Abstract

Recently, Spatial Frequency Domain Imaging (SFDI) has gradually become an alternative method to extract tissue optical properties (OPs), as it provides a wide-field, no-contact acquisition. SFDI extracts OPs by least-square fitting (LSF) based on the diffuse approximation equation, but there are shortcomings in the speed and accuracy of extracting OPs. This study proposed a Long Short-term Memory Regressor (LSTMR) solution to extract tissue OPs. This method allows for fast and accurate extraction of tissue OPs. Firstly, the imaging system was developed, which is more compact and portable than conventional SFDI systems. Next, numerical simulation was performed using the Monte Carlo forward model to obtain the dataset, and then the mapping model was established using the dataset. Finally, the model was applied to detect the bruised tissue of ‘crown’ pears. The results show that the mean absolute errors of the absorption coefficient and the reduced scattering coefficient are no more than 0.32% and 0.21%, and the bruised tissue of ‘crown’ pears can be highlighted by the change of OPs. Compared with the LSF, the speed of extracting tissue OPs is improved by two orders of magnitude, and the accuracy is greatly improved. The study contributes to the rapid and accurate extraction of tissue OPs based on SFDI and has great potential in food safety assessment.

## 1. Introduction

The study of the propagation process of light in biological tissue has been a hot issue. It has been found that tissue optical properties (OPs) show great potential in biomedical detection [1,2], OPs’ detection of fruit [3,4] and OPs’ detection of milk [5]. The propagation behavior of light in biological tissue consists mainly of absorption and scattering, which are generally quantitatively described by the absorption coefficient ($\mu_{a}$) and the reduced scattering coefficient (${\mu^{\prime }}_{s}$). The $\mu_{a}$ reflects the chemical composition of biological tissue, whereas the ${\mu^{\prime }}_{s}$ reflects the physical structural properties of the tissues [6]. Therefore, obtaining $\mu_{a}$ and ${\mu^{\prime }}_{s}$ of biological tissue is important for assessing the physicochemical properties of biological tissue. There are various methods to obtain tissue OPs, such as the temporally resolved [7], spatially resolved [8], and integrating sphere methods [9]. As a new method to obtain tissue OPs, Spatial Frequency Domain Imaging (SFDI) is widely used in burned tissue assessment [10], meat classification [11], and bruised fruit detection [12,13]. The SFDI technique is commonly used in the biomedical field, but it is rarely used in food safety evaluation and agricultural product quality assessment.

There are two homogeneous forward models of mapping from OPs to diffuse reflectance in Spatial Frequency Domain Imaging. One model is an analytic approach based on the diffusion approximation equation and another model is based on transport using Monte Carlo (MC) simulations [14,15]. The main task of extracting tissue OPs by transport models is to deal with an inverse process of mapping tissue OPs to spatial frequency diffuse reflectance. There are two ways to implement the inversion process, one is the error minimization, and the other is the search method. For the first approach, the error minimization problem ($\min\sum \left(R_{d,\mathrm{model}}\left(f_{x}\right){\;-\;R}_{d,\mathrm{sample}}\left(f_{x}\right)\right)$) is solved by inputting a guess value of the optical properties into the model to obtain the diffuse reflectance ($R_{d,\mathrm{model}}$) closest to the actual value ($R_{d,\mathrm{sample}}$). The second approach is a search problem, which first generates a large amount of data using a forward model and then compares the diffuse reflectance of the sample and dataset to find the optical properties values. Regardless of which forward model is used, there are two common methods used for inversion so far. One is the least-square fitting (LSF) method, and the other is the look-up table (LUT) method [16,17]. Generally, to obtain accurate and stable results, diffuse reflectance at multiple spatial frequencies is used [18,19]. However, whether using analytic approach based on the diffusion approximation equation or MC simulations based on transport, the LSF is computationally slow and unsuitable for fitting large numbers of pixels, which is an inherent drawback of the fitting method. The LUT method generates a diffuse reflectance dataset from a forward model and then builds a mapping table from diffuse reflectance to OPs, and the inversion process usually uses interpolation to estimate the OPs. In theory, if the interval of the LUT is small enough, extremely high inversion accuracy can be obtained. However, with the decrease of LUT interval and the increase of frequency number, the inversion time increases exponentially. The LUT therefore requires a compromise between accuracy and speed. In conclusion, traditional inversion methods are not good to balance accuracy and speed at the same time. Therefore, it is necessary to improve the speed and accuracy of SFDI inversion to quantify the OPs of tissue quickly and accurately.

Machine learning is widely used in visual inspection [20], quality assessment of agricultural products [21], and metal material research [22]. Since machine learning techniques have great advantages in dealing with regression problems with large amounts of data, they are used to replace the time-consuming model-based inversion process in diffuse reflectance optics [23,24]. The mapping between OPs and diffuse reflectance is strongly nonlinear in SFDI. Meanwhile, machine learning and regression techniques were found to be highly advantageous in solving nonlinear problems; for example, an artificial neural network (ANN) implementation for extraction of tissue OPs [25], and extraction of tissue OPs based on random forest regressor (RFR) [26]. According to the literature [27,28], machine learning-based extraction of OPs can be two orders of magnitude faster than conventional methods, without degrading the accuracy of OPs, based on the SFDI technique. Although these methods are based on machine learning, which greatly improves the prediction speed, the prediction accuracy is still lacking.

The analysis of OPs allows for the assessment of physiological indicators such as firmness and Soluble Solids Content (SSC) [6], which helps in the evaluation and classification of fruits. Fruits are prone to receive crushing and bruising during the picking, transportation, and marketing process. Over time, the bruised tissues of pears will decay and spread to the surrounding tissue, which eventually leads to a decrease in the economic efficiency of pears. Furthermore, it is a good mean to detect the bruised tissue of fruits by Ops. Therefore fast, accurate, and portable extraction of tissue OPs is of great importance in agricultural production and food safety.

Researchers have been looking for fast and accurate inversion methods, aiming to achieve real-time, accurate, and portable extraction of tissue OPs based on the SFDI technique. Common mapping models based on machine learning methods are used to extract OPs, which greatly improve the prediction speed and prediction accuracy. However, accuracy is still lacking. In this study, a mapping method based on Long Short-term Memory (LSTM) [29] was proposed to extract OPs, which is not only fast, but also improves accuracy. This work lays a foundation for solving the problem of real-time, accurate, and portable extraction of tissue OPs based on the SFDI technique. The purpose of this study was to look for an alternative approach to extract OPs quickly and accurately from diffuse reflectance images for bruised tissue detection in ‘crown’ pears. Therefore, the main objectives of this research are as follows: (1) build a compact and portable system; (2) obtain data through Monte Carlo simulation; (3) establish a mapping model; and (4) detect the change of tissue OPs after a ‘crown’ pear has been bruised.

## 2. Materials and Methods

### 2.1. Spatial Frequency Domain Imaging Instrumentation

**Figure 1:**
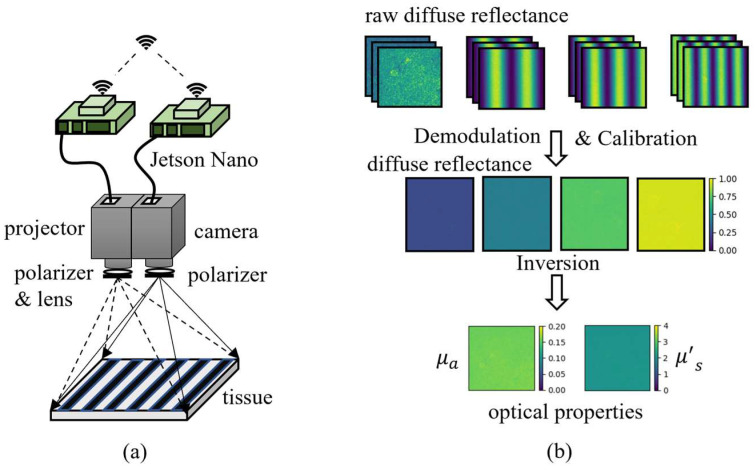
(**a**) for SFDI instrumentation and (**b**) for data processing.

The Spatial Frequency Domain Imaging system is shown in Figure 1a. The grayscale illumination pattern is generated by a miniature projection module. In this study, we used a digital projector (M1, Lenovo, Beijing, China), based on a digital micromirror-based digital light processing (DLP) light engine (Texas Instruments, Dallas, TX, USA) and an LED light source. A filter (λ = 525 nm, Δλ = 10 nm, Beijing Optical Century Instrument co. LTD (BOCIC), Beijing, China) was used in front of the lens of the projector to filter out light with a wavelength of 525 nm. The diffuse light reflected from the sample surface was captured by an 8-bit CCD camera (MV-CA060-11GM, Hikvision, Hangzhou, China). The generation of the illumination pattern, the projection, and the acquisition of the diffuse reflectance image of the sample were implemented by two ARM boards (Jetson Nano, Nvidia Corporation, Santa Clara, CA, USA). The synchronization of the sinusoidal illumination pattern projection and the sample diffuse reflectance acquisition was ensured by network communication. Compared to conventional systems, this study abandoned the strategy of using a personal computer as the control core and used miniature components, making the system more portable and compact.

### 2.2. SFDI Processing

For the Spatial Frequency Domain Imaging technique, the sinusoidally modulated light is projected onto the surface of the scattering medium first, and then the raw diffuse reflectance image is captured with a camera. Sinusoidally modulated light at each frequency needs to be projected three times with the phase 0π, 2π/3, and 4π/3. At least two frequencies are required to map the optical properties (OPs) using the optical transport model [30]. The data processing is shown in Figure 1b.

After obtaining the raw diffuse reflectance image, the modulation amplitude (${M(f}_{x})$) needs to be obtained by three-phase demodulation, as in Equation (1).

(1) $$M\left(f_{x}\right)=\frac{\sqrt{2}}{3}{{\{(I}_{1}\left(f_{x}\right){\;-\;I}_{2}\left(f_{x}\right){)}^{2}{+(I}_{2}\left(f_{x}\right){\;-\;I}_{3}\left(f_{x}\right){)}^{2}{+(I}_{3}\left(f_{x}\right)\;{-\;I}_{1}\left(f_{x}\right){)}^{2}\}}^{1/2}$$

where $I_{i}{(f}_{x})$ is the raw diffuse reflectance with different phases at the same frequency, and ${M(f}_{x})$ is the modulation amplitude. The correction is then performed using a reference whiteboard with known diffuse reflectance, as in Equation (2),

(2) $$R_{d}{\left(f_{x}\right)}_{\mathrm{measured}}=\frac{M{\left(f_{x}\right)}_{\mathrm{measured}}}{M{\left(f_{x}\right)}_{\mathrm{reference}}}R_{d}{\left(f_{x}\right)}_{\mathrm{reference}}$$

where $M{\left(f_{x}\right)}_{\mathrm{measured}}$ is the modulated amplitude of the sample, $M{\left(f_{x}\right)}_{\mathrm{reference}}$ is the modulated amplitude of the reference whiteboard, $R_{d}{\left(f_{x}\right)}_{\mathrm{measured}}$ is the diffuse reflectance of the sample, and $R_{d}{\left(f_{x}\right)}_{\mathrm{reference}}$ is the diffuse reflectance of the reference whiteboard. According to the diffuse approximation theory, the optical properties ($\mu_{a}$, $\mu^{\prime }{}_{s}$) can be solved using curve fitting based on the diffuse equation [30] after the diffuse reflectance is obtained. This is shown in Equation (3),

(3) $$R_{d}\left(f_{x}\right)=\frac{{{3A\mu }^{\prime }}_{s}{/\mu }_{\mathrm{tr}}}{\left({\mu \;}_{\mathrm{eff}}^{\prime }/\mu_{\mathrm{tr}}+1\right)\left({\mu \;}_{\mathrm{eff}}^{\prime }/\mu_{\mathrm{tr}}+3A\right)}$$

where $\mu_{\mathrm{tr}}{=\mu }_{a}{+\mu }^{\prime }{}_{s}$ is the transport coefficient, $\mu_{\mathrm{eff}}^{\prime }=\sqrt{{3\mu }_{a}\mu_{\mathrm{tr}}{+2\pi f}_{x}{}^{2}}$ represents the scalar attenuation coefficient in the spatial frequency domain, n is the refractive index of sample, $R_{\mathrm{eff}}=0.63n+0.668+0.71/n\;-\;1{.44/n}^{2}$ is effective reflection coefficient, and ${A=(1\;-\;R}_{\mathrm{eff}}{)/2(1+R}_{\mathrm{eff}})$ is a proportionality constant.

### 2.3. Monte Carlo Simulations

Unlike the diffuse approximation equation, Monte Carlo (MC) simulation is a stochastic statistical method that simulates the transport of photons through tissue. After the photons enter the tissue, they constantly interact, and some of the photons are absorbed and disappear. Photons emitted from the upper surface of the tissue form diffuse light, and photons emitted from the lower surface of the tissue form transmitted light. Given the optical properties (OPs) parameters, the purpose of MC simulations is to simulate the photons transport process and then accurately calculate the corresponding diffuse reflectance. Many researchers have implemented MC simulations programs for different purposes, some for time-domain MC simulations [31], some for single-layer tissue MC simulations, and some for multi-layer tissue MC simulations [32]. This study uses a GPU-accelerated simulations program developed by Eric [33]. A large amount of mapping data (from OPs to diffuse reflectance) was obtained through MC simulations, which was used to construct the Long Short-term Memory Regressor model.

Given the value of the OPs, the diffuse reflectance $R_{d}\left(r\right)$ can be obtained using the MC simulations program. However, this spatially distributed diffuse reflectance $R_{d}\left(r\right)$ obtained by MC simulations is independent of the frequency of the structured light. The diffuse reflectance $R_{d}\left(f_{x}\right)$ in the spatial frequency domain (SFD) can be derived by Fourier transform [30]. As shown in Equation (4),

(4) $$R_{d}\left(f_{x}\right)=2\pi \overset{n}{\underset{i=1}{\sum }}r_{i}J_{0}{(2\pi f}_{x}r_{i}{)R}_{d}{(r}_{i})\Delta r_{i}$$

where $R_{d}\left(f_{x}\right)$ is diffuse reflectance in SFD, $r_{i}$ is the radial distance of the ith photon from the incident point of the light source in the MC simulation, $f_{x}$ is the frequency of the sinusoidally modulated light, $R_{d}{(r}_{i})$ is the reflection weight of the photon at the point $r_{i}$, $\Delta r_{i}$ is the distance between radially adjacent photons, and $J_{0}$ is the zeroth-order Bessel function of the first kind. The initialization parameters of MC simulations are shown in Table 1.

### 2.4. Long Short-Term Memory Regressor Method

The Long Short-term Memory (LSTM) network model has great potential to solve problems where input sequences have context relations. Meanwhile, the LSTM network model performs very well in solving complex nonlinear problems. Therefore, this study decided to build a mapping model based on a LSTM network model for mapping tissue optical properties (OPs) from diffuse reflectance.

**Figure 2:**
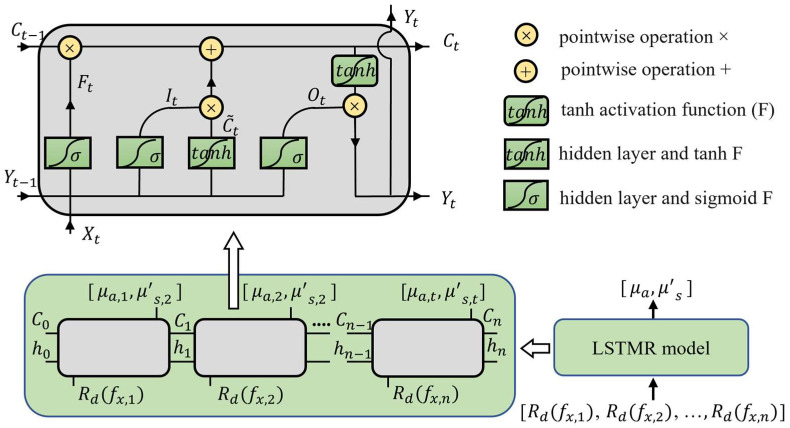
The Long Short-term Memory Regressor Model.

The Long Short-term Memory Regressor (LSTMR) model takes the n-dimensional diffuse reflectance vector and maps it to a 2-dimensional OPs vector. The input of the LSTM network is generally an n-dimensional vector with dimensions from 1 to n representing n moments. The LSTM has a memory cell that records the memory of each moment. Furthermore, the operations at each moment include adding memory and deleting memory to extract the relevant details of the context. The structure of the LSTMR model is shown in Figure 2, where the n-dimensional vector is the diffuse reflectance at different frequencies. The model of the network could be a deep neural network, and only one layer of the neural network is shown in the figure. The basic structure of the model is shown in the upper of Figure 2, and equations are shown in Equations (5)–(10),

(5) $$F_{t}{=\sigma (W}_{f}\left[Y_{t-1}{,X}_{t}\right]+z_{f})$$

(6) $$I_{t}{=\sigma (W}_{i}\left[Y_{t-1}{,X}_{t}\right]+z_{i})$$

(7) $$\;\tilde{C_{t}}{=\tan h(W}_{c}\left[Y_{t-1}{,X}_{t}\right]+z_{c})$$

(8) $$O_{t\;}{=\sigma (W}_{o}\left[Y_{t-1}{,X}_{t}\right]+\;z_{o})$$

(9) $$C_{t}{=F}_{t}⊙C_{t-1}{+I}_{t}\;⊙\;\tilde{C_{t}}$$

(10) $$Y_{t}{=\tan h(F}_{t}\;⊙{\;C}_{t-1}{+I}_{t}⊙\tilde{C_{t}})\;⊙{\;O}_{t}$$

where ⊙ is the pointwise multiplication operation, σ is the sigmoid function, W is the weight matrix of the network layer, z is the bias term of the network layer, $F_{t}$ is the forget gate, =$I_{t}$ is the input gate, $\tilde{C_{t}}$ is the current memory, $O_{t}$ is the output gate, $C_{t}$ is the memory cell at moment t, $h_{t}$ is the output at moment t, and $X_{t}$ is the input at moment t. LSTM modifies the content of the memory cell through all the forget and input gates to extract context-related information. The final output of the model can be written as Equation (11),

(11) $$\left[\mu_{a},{\mu^{\prime }}_{s}\right]=\sum w_{t}Y_{t}$$

where $w_{t}$ is the weight of the output corresponding to each component of the input vector, and $\mu_{a}$ and $\mu^{\prime }{}_{s}$ are the OPs.

The Long Short-term Memory Regressor model was constructed based on the PyTorch framework (PyTorch, version 1.10.0+cu113, Meta, Menlo Park, CA, USA), which is a mainstream framework for building machine learning models. To obtain reliable and stable models, five-fold cross-validation was used in the optimization of model parameters. Theoretically, if there are enough nodes, an artificial neural network with one hidden layer can fit any complex function. This study obtained the best results when the number of nodes was 2^5^ and the number of hidden layers was 5. Too small a batch size will lead to model oscillation and difficult convergence. Setting the batch size to the training set size is a good choice due to the small dataset, and choosing the Resilient Propagation optimizer, which is preferred when the batch size is equal to the training set size, has proven to be a smart choice. When the initial learning rate of the model was 0.0001, the model had a good convergence effect, and the model converged quickly before 200 epochs. Finally, the dropout algorithm was used to prevent overfitting, and the final model training took 30 min.

### 2.5. Model Testing

#### 2.5.1. Simulations Experiments

The Long Short-term Memory Regressor (LSTMR) model was tested using a simulated dataset, and the tested dataset never appeared in the training dataset. To accelerate the inversion speed, the two-frequency inversion strategy is usually adopted. In this study, there were six alternative frequencies ($f_{x}$ = 0.167, 0.180, 0.200, 0.220, 0.250, 0.300 mm^−1^). Different mapping models were built with different high frequencies, and a five-fold cross-validation was used in the model-building process. Different models were used to map the optical properties (OPs), and then the mean absolute error of OPs was used as the basis for the preference.

After determining the optimal frequency, the full set of training dataset was used to train the Long Short-term Memory Regressor mapping model. To highlight the advantages of the model, least-square fitting (LSF), artificial neural network (ANN), random forest regressor (RFR), and recurrent neural networks (RNN) mapping methods were implemented in the experiments, respectively. The strengths and weaknesses of the models were evaluated by the normalized mean absolute error (NMAE), the determination coefficient (R^2^), the root mean square error (RMSE), and the mean absolute error (MAE). Look-up table inversion was not chosen because it required a tradeoff in time and accuracy.

#### 2.5.2. Phantoms Experiments

By using Indian ink (Royal Talens, Apeldoorn, The Netherlands) as an absorbing agent and titanium dioxide (T104950-500 g, Aladdin Biochemical Technology Corporation., Shanghai, China) as a scattering agent, deionized-water-based optical phantoms were prepared. Five gradients were set for absorption and reduced scattering, respectively. The volume fraction of India ink is 0.006–0.014% with a 0.002% interval and the volume fraction of TiO_2_ is 0.04–0.12% with a 0.02% interval. Twenty-five small liquid phantoms with various optical properties (OPs) were fabricated. The absorption range of these phantoms was from 0.0945 mm^−1^ to 0.1905 mm^−1^, and the scattering range was from 1.3689 mm^−1^ and 4.1063 mm^−1^. According to Lambert’s law, the standard value of the absorption coefficient was derived using the collimated transmittance T, and the collimated transmittance T is obtained by spectrometer acquisition (QE65pro, Ocean Insight Corporation, Orlando, FL, USA), as shown in Equation (12),

(12) $$\mu_{a}=\frac{-\ln\left(T\right)}{D}=\frac{-\ln\left(I/I_{0}\right)}{D}$$

where D is the length of the optical path passed in the liquid during collimated transmission, and $I_{0}$ and I are the transmitted light intensity of water and the transmitted light intensity of the absorber, respectively. The standard value of the reduced scattering coefficient can be calculated by using the Mie program [34] given the parameters of TiO_2_, the refractive index of water, and the wavelength of light. The parameters of TiO_2_ include diameter, volume fraction, and refractive index.

The phantom experiments were performed using a two-frequency strategy (high frequency is the optimal frequency 0.25 mm^−1^) and an inversion was performed using the Long Short-term Memory Regressor (LSTMR), the least-square fitting (LSF), the artificial neural network (ANN), the random forest regressor (RFR), recurrent neural network (RNN), respectively. A 300 × 300-pixel area near the center pixel of each phantom was selected as the target area and the OPs images were computed.

#### 2.5.3. Pear Experiments

‘Crown’ pears were selected as experimental objects, and all crown pears came from fruit supermarkets. The surface of these pears was not damaged, and they were very fresh. The experiments were conducted in March at an ambient temperature of 20 degrees Celsius. During the bruising treatment, the pendulum motion was simulated by using a small iron ball to hit the pear around the equator, thus inducing the formation of bruised tissue. During the experiments, the experimental subjects were consistent before and after bruising, and the images of normal pears were collected first, and then the images of bruised pears were collected after bruising treatment. All experimental procedures used the same system to acquire images and the same program to extract optical properties.

## 3. Results

### 3.1. Simulation Experiment Results

**Figure 3:**
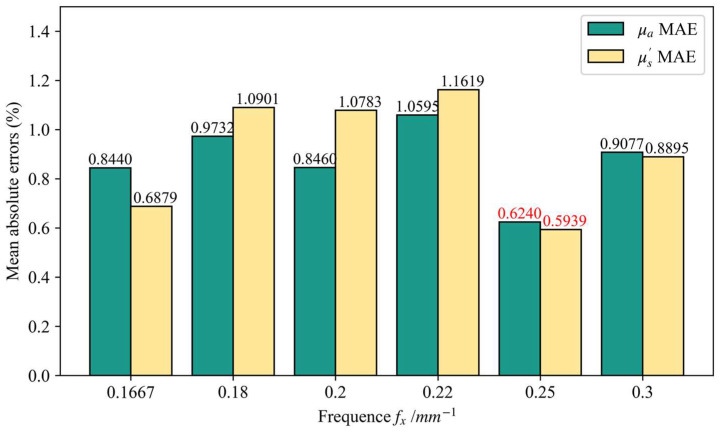
The Mean absolute errors of different models in predicting optical properties. The horizontal coordinate is the mapping model for different high frequencies. The numbers marked in red are the minimum mean absolute errors.

To obtain the best high frequency, the mapping models with different high frequencies were built based on the training dataset, and the most suitable high frequencies were determined in the range of 0.167–0.300 mm^−1^. Figure 3 illustrates the mean absolute error (MAE) of the optical properties (OPs), where the horizontal axis is the mapping model for different high frequencies. The results show that the model has the best accuracy when the frequency is chosen to be 0.25 mm^−1^, and when the MAE of the absorption coefficient ($\mu_{a}$) and the reduced scattering coefficient ($\mu^{\prime }{}_{s}$) are 0.6240% and 0.5939%, respectively. The optimal frequency of 0.25 mm^−1^ is very close to the commonly used optimal frequency of 0.2 mm^−1^, which is consistent with the experimental results of Luo’s frequency preference [5].

**Figure 4:**
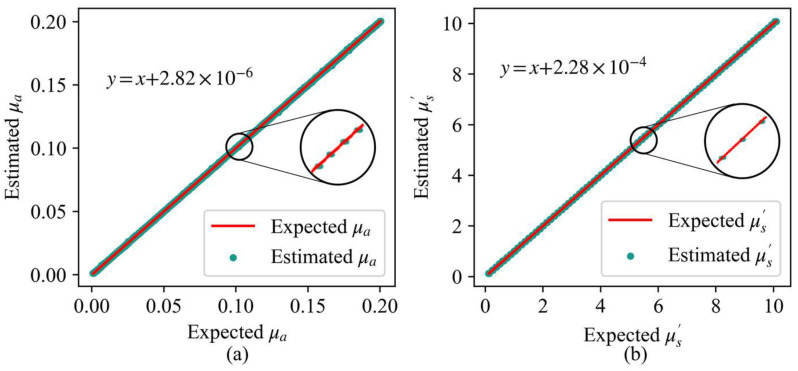
Linearity between the measured and calculated values of the optical properties in the simulation experiments, (**a**) is for the absorption coefficient and (**b**) is for the reduced scattering coefficient.

The prediction results of different models are shown in Table 2. The prediction results of the Long Short-term Memory (LSTMR) are optimal in terms of normalized mean absolute error (NMAE), MAE, root mean square error (RMSE), and determined coefficient (R^2^). Except for the LSTMR model, the RFR model has the best prediction results. For LSTMR, the MAE of the $\mu_{a}$ and the $\mu^{\prime }{}_{s}$ are 0.32% and 0.21%, respectively. This is an order-of-magnitude improvement compared to the prediction accuracy of the LSF. As shown in Figure 4, there is an extremely high linearity between the predicted and target values of the LSTMR, with the R^2^ approaching 1. As can be seen in Figure 4, the target and predicted values almost exactly coincide and overlap in a straight line, both for the $\mu_{a}$ and the $\mu^{\prime }{}_{s}$. This indicates that the model is an excellent fitting and that the model fits well as a function of the diffuse reflectance and OPs. The experiments illustrate that LSTMR is an ideal model for accurately mapping OPs.

### 3.2. Phantoms Experiments Results

To verify that the proposed Long Short-term Memory Regressor (LSTMR) mapping model can be used to extract optical properties (OPs) accurately and quickly, 25 optical phantoms with known OPs were produced. As shown in Table 3, LSTMR mapped OPs at a speed of 253 ms for a 300 × 300-pixel image (CPU, Intel-I7-11800H). However, for the least-square fitting (LSF) method, extracting the OPs of a 300 × 300-pixel image took 57,970 ms. The results show that the LSTMR inversion speed is improved by 2 to 3 orders of magnitude compared to LSF. The speed of predicting tissue OPs based on machine learning methods depends on the complexity of the model (number of nodes and number of network layers), so this study only implemented a speed comparison between LSTMR and LSF. 

**Figure 5:**
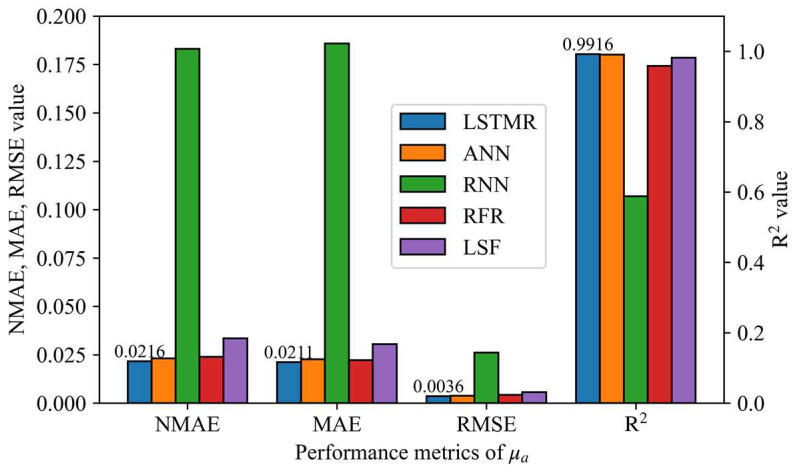
Performance metrics for different models in the phantom experiment for absorption coefficient.

**Figure 6:**
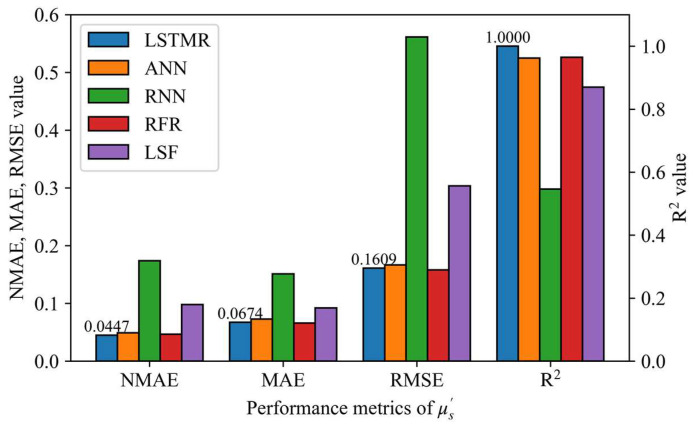
Performance metrics for different models in the phantom experiment for reduced scattering coefficient.

The results of different inversion methods for the phantoms experiments are shown in Figure 5 and Figure 6, and the mean absolute error (MAE) of $\mu_{a}$ and $\mu^{\prime }{}_{s}$ are 0.0211 and 0.0674 using the LSTMR method, respectively. Furthermore, the R^2^ of $\mu_{a}$ and $\mu^{\prime }{}_{s}$ are 0.9916 and 1.0, respectively, which indicates that the predicted results of LSTMR have a good linear relationship with the expected values. It confirms that LSTMR is an ideal choice for inversion in Spatial Frequency Domain Imaging. Due to the inevitable experimental error, the actual value of the phantom is different from the reference value, so the prediction result of the phantom would be slightly worse than the simulation result. The relative error of $\mu^{\prime }{}_{s}$ is larger than that of $\mu_{a}$ because the scattering agent is easily precipitated and is more influenced by whether the liquid surface is stationary or not, resulting in a larger error in the prediction of ${\mu^{\prime }}_{s}$. Obviously, the mapping results of the LSTMR inversion model are better than other models in the experiments.

### 3.3. Pear Experiment Results

**Figure 7:**
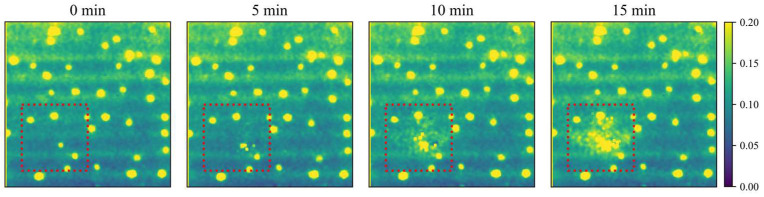
Changes in the absorption coefficient of bruised tissue of ‘crown’ pears.

**Figure 8:**
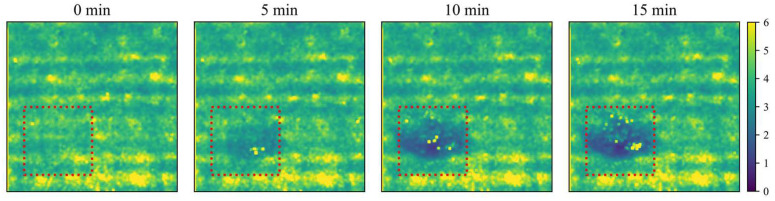
Changes in the reduced scattering coefficient of bruised tissue of ‘crown’ pears. The red box indicates changes in tissue optical properties in the bruised area of the pear.

The results of the bruised tissue detection experiment for pears are shown in Figure 7 and Figure 8. Pears will form bruised tissue at an early stage after being slightly crushed. In this study, it was demonstrated that bruised tissue forms on the surface of the pear after a slight impact. The absorption coefficient of the tissue increases during the formation of bruised tissue. The opposite is true for the reduced scattering coefficient, which is consistent with the experimental results of Sun [25] and Luo [35]. Moreover, both absorption and reduced scattering images could highlight the areas of bruised tissue. Therefore, using the Spatial Frequency Domain Imaging technique, early bruised detection of fruits can be performed. Furthermore, it can effectively control the bruised of fruits during transportation, thus controlling the cost of the fruit industry. The optical properties (OPs) of apple tissues can be used for nondestructive quality or ripeness prediction of apples [4], and the Long Short-term Memory method proposed in this study can obtain prediction results more accurately and quickly. Therefore, the rapid acquisition of OPs in tissues is of particular importance. This further illustrates the need to improve the speed and accuracy of extracting the OPs of tissues. It also lays the foundation for the real-time, portable acquisition of tissue OPs.

## 4. Discussion

Absorption and scattering have different sensitivities to frequency. Absorption is mainly sensitive to low frequency, whereas scattering is mainly sensitive to high frequency. The non-zero frequency should not be too large or too small for the two-frequency inversion. The simulations experimental results showed that $f_{x}$ = 0.25 mm^−1^ was the most suitable frequency under these experimental conditions, which was also closer to the non-zero frequency used in the existing literature [5]. The simulations experimental results show that the mapping accuracy of the Long Short-term Memory Regressor (LSTMR) model could be substantially improved, and the mean absolute error (MAE) of $\mu_{a}$ and $\mu^{\prime }{}_{s}$ could reach 0.32% and 0.21%, respectively. The mapping accuracy of LSTMR is much better than that of the traditional LSF method, and it also performs better than the other machine learning methods in the experiments. Compared with the LSF method, the phantoms experiment not only shows that LSTMR has an advantage in mapping accuracy, but also has a huge performance advantage in inversion speed.

Jäger et al. [23] combined spatial resolution technology with multiple artificial neural network to extract optical properties (OPs). According to their reports, the normalized mean absolute errors (NMAEs) of $\mu_{a}$ and $\mu^{\prime }{}_{s}$ are 6.1% and 2.9%, respectively. The MAE of the deep neural network mapping model proposed by Stier [1] for $\mu^{\prime }{}_{s}$ is 6.8%. Song [28] developed an OPs mapping model based on the deep neural network, and according to their study, the mean and standard deviation of the percentage errors of $\mu_{a}$ and $\mu^{\prime }{}_{s}$ were 0.0 ± 1.4 and 0.0 ± 0.28%, respectively. Sun proposed an artificial neural network method [25] for the inversion of OPs based on multi-frequency inversion, where the NMAEs of $\mu_{a}$ and $\mu^{\prime }{}_{s}$ are 0.18% and 0.027%, respectively. Sun used seven frequencies for inversion, whereas we used only two frequencies to achieve comparable accuracy. Panigrahi [26] demonstrated that the random forest regressor (RFR) method was a highly accurate and fast inversion method, and the MAE of OPs could be reduced to 0.556% and 0.126%, respectively. Comparing the MAE, it could be found that the $\mu_{a}$ of LSTMR was more accurate, whereas the $\mu^{\prime }{}_{s}$ of RFR was more accurate. The $\mu^{\prime }{}_{s}$ of LSTMR is slightly less accurate than RFR due to the large gradient (the interval of $\mu^{\prime }{}_{s}$ is 0.126 mm^−1^) of the ${\mu^{\prime }}_{s}$ of the dataset.

The phantom experimental results showed that the LSTMR not only has better inversion accuracy than other methods, but also had a dramatic improvement in inversion speed, with a speed improvement of 2 to 3 orders of magnitude compared to the LSF. The LSF method requires continuous iterations for optimization until the error is within an acceptable range, which consumes a lot of time during the iterations, and which is evident as the number of frequency increases. The look-up table uses a search strategy in which the time taken for the search process increases exponentially as the number of frequencies increases. However, using multiple spatial frequencies for inversion can improve the robustness of the model [18]. The machine learning method can solve the slow speed problem in the process of multi-frequency inversion, and the mapping accuracy can be improved at the same time. As can be seen from Table 3, LSTMR is more than 100 times faster than LSF. This is also consistent with the results of Zhao’s study [27] and Song’s study [28].

## 5. Conclusions

The proposed Long Short-term Memory Regressor (LSTMR) method is an ideal mapping model to replace the inversion method based on the optical transport model. It can quickly extract optical properties (OPs), but without loss of estimation accuracy. This study not only compared the LSTMR method to the traditional LSF method, but also to other machine learning methods that appeared in journals, and it turns out that the LSTMR method is indeed a good choice. The experimental results show that the accuracy of LSTMR inversion is comparable to or even better than that of the previous literature. Furthermore, the speed of LSTMR is improved by 2~3 orders of magnitude compared with LSF. These pear experiments proved that LSTMR can accurately distinguish bruised tissue, which provides a feasible solution for the quality assessment of pears. All experiments are based on our developed miniaturized Spatial Frequency Domain Imaging system. This study laid the hardware foundation and method foundation for real-time and portable OPs acquisition of pears, and further applied it to pear quality evaluation.

## Figures and Tables

**Table 1:** Parameter settings for the Monte Carlo simulations.

Parameters	Symbols ^1^	Value
Number of photons	N	3,000,000
Resolution	dz ^2^, dr ^3^	0.01 cm
Anisotropy	g	0.7
The thickness of tissue	d	5 cm
Refractive index air	*n*_0_	1
Refractive index of tissue	*n*_1_	1.34

^1^ The symbols corresponding to the parameters. ^2^ The thickness of each tissue layer in the Monte Carlo simulations. ^3^ The thickness of each tissue layer in the radial direction of the light source.

**Table 2:** Predictive performance of different mapping models in simulation experiments.

OPs	Metric	LSTMR	ANN	RNN	RFR	LSF
$\mu_{a}$	NMAE	0.0012	0.0069	0.0077	0.0049	0.0506
	MAE	0.0032	0.0151	0.0181	0.0171	0.0597
	RMSE	0.0002	0.0010	0.0012	0.0007	0.0207
	R^2^	1.0000	0.9999	0.9999	0.9999	0.9999
$\mu^{\prime }{}_{s}$	NMAE	0.0009	0.0046	0.0053	0.0036	0.0598
	MAE	0.0021	0.0127	0.0139	0.0023	0.0770
	RMSE	0.0060	0.0298	0.0350	0.0226	0.3323
	R^2^	1.0000	0.9971	0.9966	0.9978	0.9996

**Table 3:** The speed of mapping from diffuse reflectance to optical properties.

Methods	Resolution/Pixel	Speed ms/Pixel
LSF	300 × 300	0.6441
LSTMR	300 × 300	0.0028

## Data Availability

The data that support the findings of this study are available from the corresponding author upon reasonable request.
